# Biodegradable Contact Lenses for Targeted Ocular Drug Delivery: Recent Advances, Clinical Applications, and Translational Perspectives

**DOI:** 10.3390/molecules30122542

**Published:** 2025-06-10

**Authors:** Iwona Rykowska, Iwona Nowak, Rafał Nowak, Ola Michałkiewicz

**Affiliations:** 1 Faculty of Chemistry, Adam Mickiewicz University, Uniwersytetu Poznańskiego 8, 61-614 Poznań, Poland; iwona.nowak@amu.edu.pl (I.N.); ola.michalkiewicz@amu.edu.pl (O.M.); 2 Department of Ophthalmology, Józef Struś City Hospital, Szwajcarska 3, 61-285 Poznań, Poland; raf.nowak@wp.pl

**Keywords:** biodegradable polymers, drug-eluting contact lenses (DECLs), ophthalmic biomaterials, sustained drug release

## Abstract

Ocular drug delivery presents a persistent clinical challenge due to the protective anatomical structure of the eye, physiological barriers such as reflex blinking, and continuous tear fluid turnover. These factors significantly limit the bioavailability of topically applied medications, reducing the therapeutic effectiveness of conventional formulations, such as eye drops, ointments, and suspensions, particularly in the management of chronic ocular disorders, including dry eye syndrome, diabetic retinopathy, and age-related macular degeneration. Drug-eluting contact lenses (DECLs) offer a promising alternative, enabling sustained, localized, and controlled drug release directly at the ocular surface. While several reviews have addressed contact lenses as drug delivery platforms, this work provides a distinct perspective by focusing specifically on biodegradable polymer-based systems. Emphasis is placed on recent advances in the design and fabrication of DECLs using natural and synthetic biodegradable polymers, which offer superior biocompatibility, customizable degradation kinetics, and the capacity for programmable drug release. This review discusses the selection criteria for polymer matrices, strategies for drug incorporation, and key factors influencing release profiles. Moreover, this study highlights innovative methodologies and therapeutic approaches that differentiate it from the existing literature, providing a timely and comprehensive resource for researchers developing next-generation polymeric ocular drug delivery systems.

## 1. Introduction

**Figure 1:**
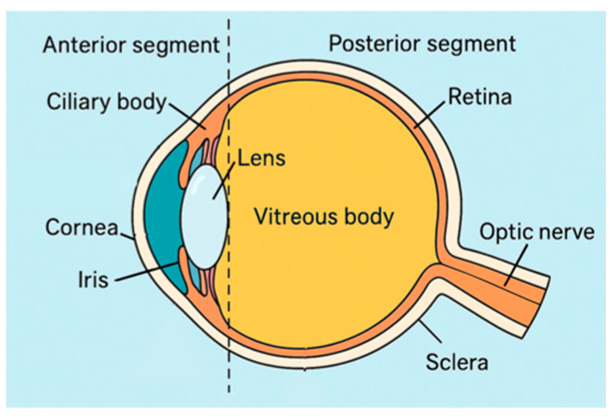
Cross-sectional diagram of the human eye showing major anatomical regions involved in ocular pharmacokinetics.

**Figure 2:**
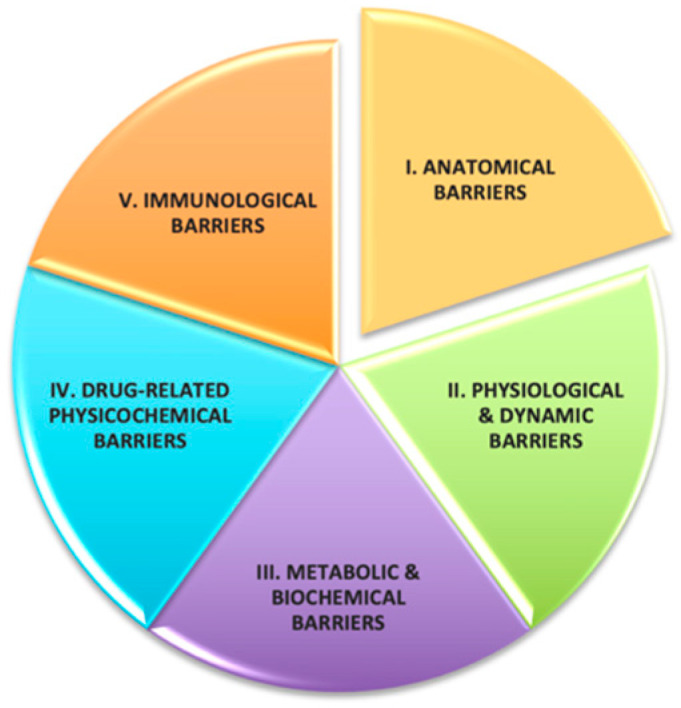
Key barriers to effective ocular drug delivery.

Effective ocular drug delivery remains a critical challenge due to the eye’s robust protective architecture and dynamic clearance mechanisms—including tear turnover, blinking, and nasolacrimal drainage—that limit drug retention and reduce therapeutic efficacy (Figure 1 and Figure 2). While eye drops remain the standard of care, they deliver less than 5% of the administered dose to intraocular tissues, necessitating frequent re-application and compromising adherence [1].

In this context, contact lenses have emerged as versatile platforms for sustained ocular drug delivery, offering improved corneal permeability, extended residence time, and site-specific release. Among these, biodegradable contact lenses constitute a particularly promising class. Engineered from natural or synthetic polymers, they undergo controlled degradation, enabling precise modulation of drug release while eliminating the need for device removal [2].

Recent research has demonstrated the potential of biodegradable lenses in delivering anti-inflammatory, anti-infective, and regenerative agents for anterior segment disorders such as keratitis, uveitis, cystinosis, and postoperative inflammation [3]. Their integration with stimuli-responsive materials, nanocarriers, and hybrid architectures—including microneedles and microfluidics—further expands their functionality [4].

This review critically synthesizes the literature from 2021 to 2025, focusing on material design, drug-loading strategies, and clinical relevance. Emphasis is placed on polymer selection, release mechanisms, and regulatory considerations shaping translational progress.

### 1.1. Modern Contact Lens Classification

**Figure 3:**
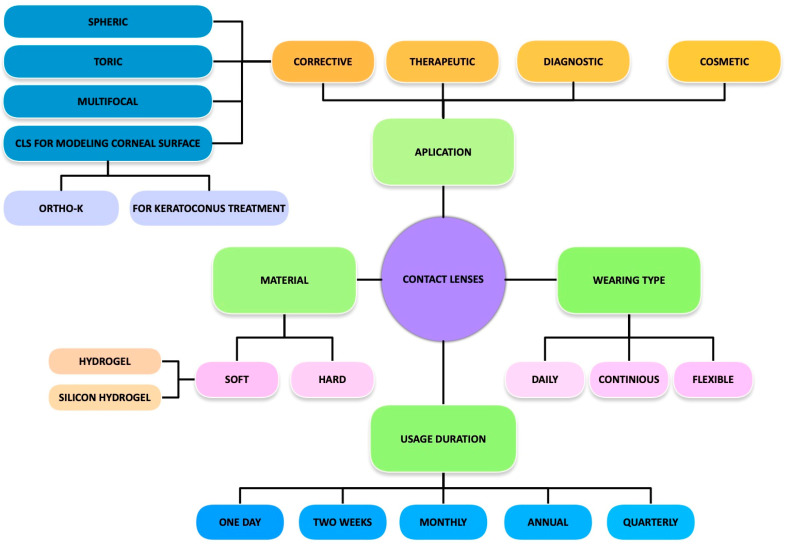
Classification of contact lenses based on material, usage duration, and application.

Modern contact lenses are now categorized by material, wearing schedule, and intended function—corrective, therapeutic, cosmetic, or specialized (e.g., orthokeratology or biosensor-integrated). An overview of this classification is presented in Figure 3 [5,6,7,8,9,10,11,12,13,14].

Despite widespread use, the complexity of therapeutic and sensor-integrated designs has prompted stricter regulatory oversight. Both the FDA and EMA classify contact lenses as medical devices; drug-eluting lenses (DECLs) are considered combination products subject to dual compliance criteria. Table 1 compares regulatory classifications and requirements in the U.S. and EU [15,16,17,18,19,20,21].

### 1.2. DECL Performance and Design Considerations

DECLs must meet high standards for pharmacokinetics, biocompatibility, and manufacturing. Their hybrid nature necessitates therapeutic consistency without compromising lens safety or optical performance. This underscores the need for interdisciplinary collaboration across pharmaceutical sciences, materials engineering, and clinical ophthalmology [22,23,24].

Drug-modified contact lenses—especially biodegradable variants—must meet stringent performance criteria: biocompatibility, optical clarity, mechanical integrity, and consistent drug release over time. Sustained, localized delivery with preserved drug stability remains a central challenge, yet promising clinical and preclinical data confirm their therapeutic value [12,25,26,27].

### 1.3. Scope and Structure of This Review

This review is organized into three major sections: (1) advances in biodegradable lens materials; (2) drug incorporation and release strategies; and (3) clinical applications and therapeutic protocols. The scope encompasses the literature from 2021 to 2025, reflecting rapid developments at the interface of polymer science, nanotechnology, and ophthalmology. A curated summary of recent review articles is provided in Table 2.

## 2. Materials and Methods

### 2.1. Materials Used in DMCLs

The development of materials for contact lenses is firmly grounded in polymer science, with particular emphasis on key properties such as hydrophilicity, biocompatibility, mechanical strength, and oxygen permeability. In addition to bulk properties, the surface characteristics of contact lenses, such as wettability, resistance to protein and lipid deposition, and influence on wearer comfort, are paramount in determining clinical performance and user satisfaction.

Recent advancements in nanotechnology and biotechnology have facilitated the emergence of technologically enhanced contact lenses, representing a new generation of multifunctional devices capable of health monitoring, controlled drug delivery, and even augmented reality integration [34,36,37,51]. These innovative systems incorporate microelectronics and embedded sensors within the lens matrix, enabling continuous intraocular pressure monitoring for glaucoma management, noninvasive glucose sensing via tear fluid for diabetic patients, and programmable medication release [51,60,61].

Other examples of advanced material solutions include UV-blocking and photochromic contact lenses. UV-blocking lenses are designed to absorb harmful ultraviolet radiation, reducing the risk of cataract formation and other phototoxic ocular conditions. Photochromic lenses, in turn, exhibit reversible darkening in response to sunlight exposure, enhancing visual comfort and protection for individuals with increased photosensitivity [51,62,63].

### 2.2. Biopolymers

Polymers constitute the foundational materials used to fabricate contact lenses, forming the structural matrix and defining the functional capabilities of conventional and drug-eluting systems. Polymeric macromolecules, composed of covalently bonded repeating monomeric units, exhibit properties fundamentally determined by their monomer’s chemical structure, the degree of polymerization, and the spatial arrangement of polymer chains [51,64]. These molecular-level features translate directly into key bulk properties—such as mechanical strength, elasticity, oxygen permeability, hydrophilicity, and biodegradability—which govern user comfort and clinical performance [51,65].

In drug-modified contact lenses (DMCLs), the choice of polymer is particularly critical, as it must support the mechanical and physiological demands of the ocular environment and enable predictable and sustained drug delivery.

Biodegradable polymers have emerged as an up-and-coming class of materials for this application because of their ability to gradually decompose under physiological conditions, reducing the need for lens removal and minimizing long-term material accumulation in ocular tissues. Their degradation profiles can be finely tuned by manipulating monomer composition, crosslinking density, and copolymer ratios—allowing precise control over drug release kinetics and lens residence time.

The development of DMCLs involves two fundamental design considerations. First, the selected polymer must be chemically and physically compatible with the intended therapeutic agent and the envisioned sterilization and storage conditions. Second, a suitable drug-loading strategy must be implemented—ranging from simple soaking techniques to advanced approaches involving molecular imprinting, nanoparticle encapsulation, or surface functionalization—to enable sustained and targeted delivery. Biodegradable polymers play a central role in these systems, as their gradual erosion can serve as a built-in mechanism for controlled drug release, often eliminating the need for additional release-modifying additives.

Integrating biodegradable polymer systems into contact lens platforms is a pivotal advancement in ophthalmic drug delivery. It offers the dual advantage of therapeutic efficacy and patient convenience, aligning material degradation with pharmacokinetic demands and contributing to safer, more sustainable, and more effective ocular therapies.

Most ophthalmic drug delivery systems rely on polymeric formulations [30,66,67]. These include a broad range of synthetic and natural biopolymers capable of functioning as drug carriers [38].

#### 2.2.1. Synthetic Biodegradable Polymers

Synthetic polymers are produced from chemically synthesized monomers. Among those approved by the FDA for ophthalmic or clinical use are poly(ethylene glycol) (PEG), poly(vinyl alcohol) (PVA), poly(glycolic acid) (PGA), poly(lactic-co-glycolic acid) (PLGA), poly[2-(dimethylamine)ethyl methacrylate] (PDMAEM), poly(caprolactone) (PCL), poly(acrylic acid) (PAA), and poly(amidoamine) (PAMAM). Many additional polymers are under preclinical investigation or approved for other biomedical applications. The monomers used to synthesize selected synthetic polymers relevant to ocular drug delivery are summarized in Table 3.

#### 2.2.2. Natural Biodegradable Polymers

In parallel, biodegradable polymers of natural origin are increasingly investigated as sustainable and biocompatible carriers for controlled ocular drug delivery. These biopolymers, derived from animal, plant, fungal, or bacterial sources, are characterized by aqueous biodegradability, low toxicity, and favorable viscoelastic properties, making them particularly attractive for ophthalmic applications [85].

Biopolymers commonly employed in ocular drug delivery include cellulose, chitosan, hyaluronic acid (HA), collagen, carboxymethylcellulose (CMC), gelatin, dextran, guar gum, pullulan, and polydopamine (PDA). Their structural repeating units are summarized in Table 4, highlighting their chemical diversity and functional versatility in the design of biodegradable contact lens platforms.

The key advantage of natural biodegradable polymers lies in their intrinsic tissue compatibility, minimal immunogenicity, and ability to form stable bio-interfaces with ocular tissues [103,104]. Hydrophilic biopolymers, such as alginate, chitosan, and gelatin, allow for the efficient encapsulation of hydrophilic drugs but may exhibit limited release duration due to their high swelling capacity and rapid diffusivity [66]. In contrast, hydrophobic synthetic polymers—including polycaprolactone (PCL), poly(lactic acid) (PLA), and Eudragit—enable extended and tunable release profiles while mitigating initial burst effects associated with surface-adsorbed drug fractions [30,66].

Both reservoir-based and matrix-type lens configurations have been studied in ophthalmic drug delivery systems. In reservoir systems, the drug is compartmentalized within a central core surrounded by a diffusion-controlling polymer barrier. In matrix systems, the therapeutic agent is uniformly distributed throughout the polymer matrix, allowing for gradual release as the material erodes or swells [30,67].

Developing innovative ocular drug delivery systems (DDSs) using biodegradable carriers with enhanced permeability and controlled release properties represents a rapidly evolving field of translational research.

A notable advancement in polymer surface functionalization was introduced by Lee et al. in 2007, who drew inspiration from marine mussel adhesion mechanisms [105]. Under mildly alkaline and aerobic conditions, dopamine—a molecule containing both catechol and amine groups—undergoes spontaneous oxidation and self-polymerization to form polydopamine (PDA), a melanin-like polymer capable of adhering to a wide range of surfaces [106].

Because of its biocompatibility, tunable surface chemistry, and low toxicity, PDA has garnered substantial interest in ocular DDSs. For example, Liu et al. demonstrated that PDA coatings on intraocular lenses (IOLs) enabled doxorubicin loading and sustained release, effectively preventing posterior capsule opacification (PCO) in animal models [107]. Jiang et al. developed PDA nanoparticles capable of loading and releasing anti-VEGF antibodies in response to oxidative stress, offering a promising strategy for age-related macular degeneration [108]. Recent studies further suggest that PDA enhances mucus penetration, supporting its application in corneal drug delivery [109].

Advances in biomaterials science have also led to the design of next-generation biodegradable polymers with tailored degradation rates, mucoadhesive behavior, and optical clarity, making them ideal for drug-loaded contact lenses, intraocular implants, and nanocarrier systems.

Although PDA has only recently been recognized as a biomaterial (since 2007), it continues to gain momentum as a versatile component in ocular platforms. For instance, Paul Demian et al. investigated the dopamine-based surface modification of silicone hydrogel contact lenses to enhance their hydrophilicity and functionality. Using a dip-coating method involving tannic acid, dopamine, and chitosan derivatives, followed by periodate oxidation and further functionalization with branched polyethyleneimine, the resulting lenses exhibited improved lipid repellency, cytocompatibility, and partial antimicrobial activity against Staphylococcus aureus [106].

These examples underscore the therapeutic potential of PDA-based systems in developing multifunctional contact lenses for sustained and responsive ocular drug delivery.

#### 2.2.3. Emerging Biodegradable Polymers

Emerging biodegradable polymers offer a versatile platform for developing next-generation ocular drug delivery systems. These materials are characterized by tailored degradation kinetics, high biocompatibility, and structural adaptability, making them suitable for integration into contact lenses, ocular implants, and nanocarrier-based systems. For instance, silk fibroin has attracted increasing attention due to its optical transparency, mechanical strength, and ability to support corneal regeneration, making it a promising candidate for therapeutic lens fabrication [110]. Similarly, poly(glycerol sebacate) (PGS), a soft elastomer with excellent flexibility and biodegradability, is under investigation as a scaffold for ocular hydrogels and implantable devices [111]. Table 5 provides an overview of natural and other emerging biopolymeric materials investigated between 2021 and 2025, summarizing their physicochemical properties, biomedical applications, and current research status.

## 3. Controlled Drug Release Strategies

A critical factor in the design of controlled drug delivery systems (CDDSs) integrated into contact lenses is the selection of an appropriate polymeric matrix. In recent years, biopolymers—either naturally derived (e.g., chitosan, gelatin, alginate) or biodegradable synthetic analogs (e.g., polylactic acid [PLA], polyglycolic acid [PGA] and their copolymers such as PLGA)—have garnered substantial interest due to their high biocompatibility, tunable degradation rates, and eco-sustainable profiles [119,120,121].

Biopolymers are structurally diverse, encompassing polysaccharides, proteins, and synthetic biodegradable polyesters. Their macromolecular architecture enables interactions with hydrophilic and hydrophobic drugs, influencing diffusion dynamics, water uptake, and degradation-mediated release. These properties can be exploited to engineer controlled drug release profiles suitable for various ophthalmic indications.

**Figure 4:**
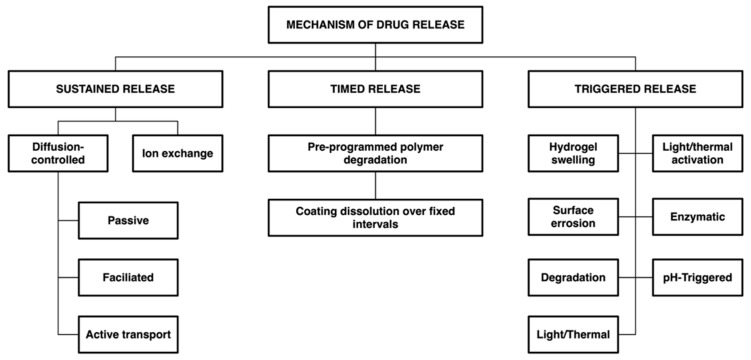
Categorization of controlled ocular drug release mechanisms.

Several major strategies have been employed in biopolymer-based drug-eluting contact lenses. Figure 4 provides a schematic representation of various drug delivery mechanisms by therapeutic contact lenses. Three principal release strategies are commonly distinguished:

**The sustained-release mechanism** involves a therapeutic agent’s continuous and prolonged diffusion over time, ensuring stable drug concentrations at the site of action. This strategy is particularly suitable for treating chronic conditions or maintaining therapeutic levels over extended periods. A prominent example of this approach is diffusion-controlled systems, in which the drug is uniformly distributed throughout a polymeric matrix and passively diffuses outward as the system interacts with the surrounding environment. Polysaccharide-based hydrogels, such as those composed of alginate or chitosan, are especially well-suited for such applications because of their high water content, structural porosity, and favorable biocompatibility [122,123].

**The timed-release mechanism** refers to drug delivery that follows a predetermined schedule, enabling administration at specific intervals that may correspond to phases of disease progression or patient-specific therapeutic requirements. A representative approach within this category is degradation-controlled systems, where the hydrolytic or enzymatic breakdown of the polymer matrix governs drug release. This mechanism is particularly applicable to synthetic biodegradable polymers, such as PLGA (poly(lactic-co-glycolic acid)) and PCL (polycaprolactone), which offer predictable erosion profiles and leave minimal residuals in biological environments [124,125].

**The triggered release mechanism** enables drug liberation in response to specific internal or external stimuli, such as temperature, pH, enzymatic activity, or light. This strategy allows for responsive, on-demand therapy, which can dynamically adapt to physiological changes in the ocular environment and improve the precision of drug delivery. Several polymer-based approaches have been developed to exploit this mechanism:

**In swelling-controlled** release, the polymer matrix swells upon exposure to tear fluid or other aqueous media, thereby increasing free volume and facilitating drug diffusion. Hydrophilic polymers, such as gelatin and cellulose derivatives, are commonly employed because of their favorable swelling behavior and biocompatibility [126].

**Layer-by-layer** (LbL) deposition is a fabrication technique involving the alternate layering of oppositely charged polymers and drug molecules, resulting in multilayered systems with tunable release kinetics. Biopolymers such as hyaluronic acid and poly(L-lysine) are frequently used for LbL assembly, enabling precise control over drug loading and sustained release from the lens surface [127].

**Molecular imprinting** is a method in which drug-specific binding sites are engineered into the polymer matrix during synthesis. This technique enhances binding specificity and allows for stimuli-responsive, sustained release, particularly when integrated with biodegradable carriers tailored for ocular applications [128].

Altogether, these strategies—particularly when implemented using biodegradable polymers—offer a powerful toolkit for developing advanced therapeutic contact lenses that meet the demands of both efficacy and ocular safety.

## 4. Drug Delivery Systems Based on Polymeric Materials

The structural diversity and tunability of biopolymers and synthetic polymers enable their widespread application as drug carriers in treating ocular diseases. These materials can be engineered into various nano- and microstructured platforms, including nanospheres, nanocapsules, liposomes, hydrogels, dendrimers, nanoparticles, nanomicelles, and microneedles.

Such nanoscale delivery systems are often integrated into larger composite matrices, including hydrogel-based contact lenses, to enhance drug loading, control release kinetics, and improve therapeutic retention on the ocular surface.

**Figure 5:**
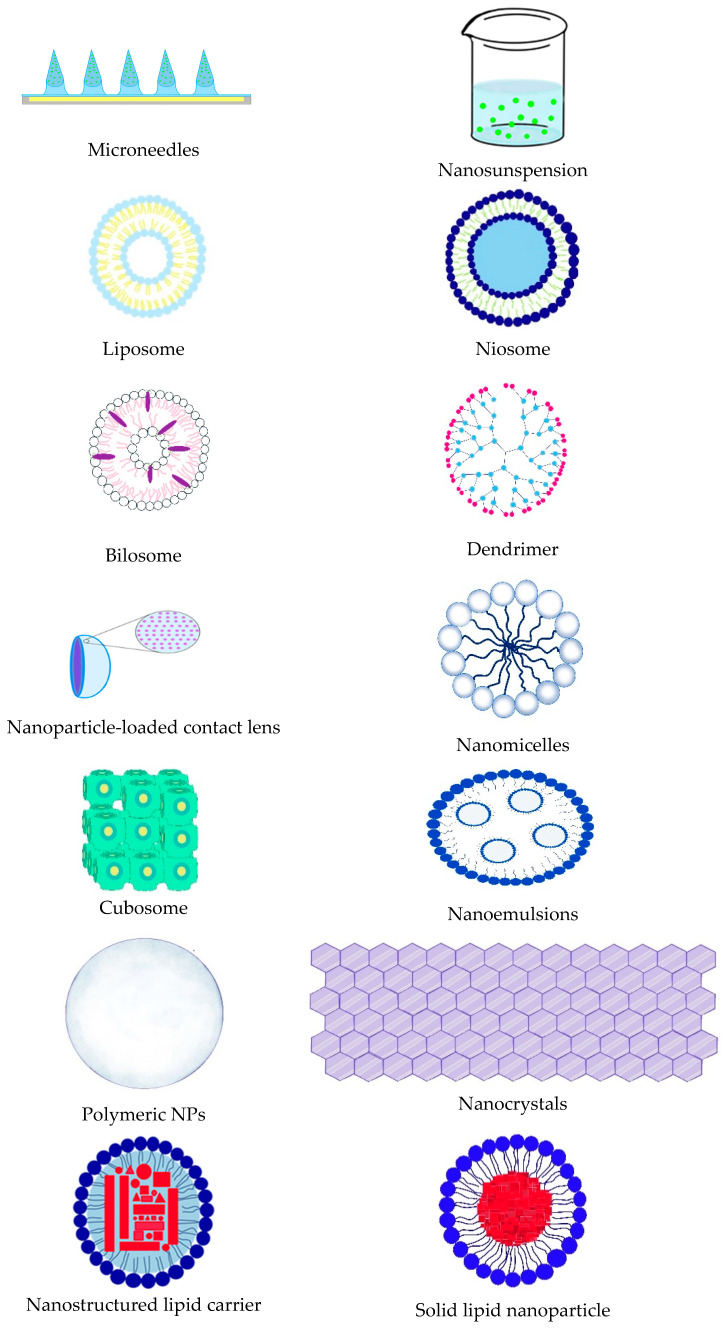
Representative polymer-based drug delivery systems for ocular administration at the macro- and nanoscale levels.

A schematic overview of polymeric drug delivery systems at both the macro- and nanoscale, currently under investigation for ophthalmic applications, is provided in Figure 5 [38,129].

## 5. Recent **Advances in Biopolymer-Based Contact Lenses**

Driven by the need for more effective, sustained, and patient-friendly ocular therapies, significant progress has been made in developing contact lens-based drug delivery systems [31,130]. These innovations integrate advanced polymeric materials with emerging nanotechnologies, enabling precise control over drug release profiles while maintaining optical clarity and wearer comfort. This section highlights selected examples of cutting-edge platforms that enhance ocular bioavailability and therapeutic efficacy.

### 5.1. Nanowafer-Based Contact Lenses

Nanowafer-based contact lenses represent a novel biodegradable and transparent ocular drug delivery platform. These disc-shaped films are fabricated from soluble polymers, such as poly(vinyl alcohol) (PVA), poly(vinyl pyrrolidone) (PVP), hydroxypropyl methylcellulose (HPMC), and carboxymethylcellulose (CMC). Within their structure, drug-loaded nano-reservoirs are embedded, enabling precisely controlled and sustained drug release over extended periods.

Owing to their resistance to physiological clearance mechanisms—including blinking and tear turnover—nanowafers achieve enhanced drug retention and ocular bioavailability [131]. Their biocompatibility, transparency, and non-irritating nature make them particularly suitable for the noninvasive delivery of protein- and peptide-based therapeutics [132].

In treating dry eye disease, nanoflakes composed of sodium carboxymethylcellulose (NaCMC) and sodium methylcellulose (NaMC) have demonstrated continuous drug release for up to 24 h. In preclinical models, dexamethasone-loaded nanoflakes showed comparable efficacy to conventional twice-daily eye drops while significantly reducing dosing frequency and improving patient compliance [133,134].

Nanowafer platforms have also shown promise in managing ocular infections and inflammatory conditions that require frequent administration. For instance, axitinib-loaded nanowafers effectively reduced both dosage and frequency in treating corneal neovascularization in murine models without inducing cytotoxic effects [135]. Similarly, cysteamine-loaded PVA nanowafers outperformed standard eye drops in treating corneal cystinosis, demonstrating improved drug stability and extended shelf life [136].

Further material advancements have been reported by Tummala et al., who developed nanocellulose-reinforced PVA hydrogel contact lenses. These materials exhibited high water content, enhanced mechanical strength, and excellent biocompatibility, all while maintaining optical clarity [137].

### 5.2. Microneedle-Based Contact Lenses

Microneedle technology represents another innovative strategy in ocular drug delivery, enabling precise and sustained therapeutic release through physical penetration of ocular barriers [138,139]. Roy et al. developed dissolvable microneedle ocular patches configured in a contact lens-like form, significantly enhancing drug bioavailability in the cornea and aqueous humor compared to conventional eye drops [140].

These systems have demonstrated the ability to deliver both low-molecular-weight compounds, such as sodium fluorescein, and macromolecular agents, including fluorescein isothiocyanate (FITC)–dextran conjugates with molecular weights up to 150 kDa [141]. To further improve retention and performance, Amer et al. introduced self-adhesive microneedle designs featuring interlocking geometries, which enable prolonged release upon contact with ocular fluids [142].

Additionally, multilayered ocular patches incorporating micro-reservoirs have been engineered for the sustained intraocular delivery of anti-VEGF agents, offering therapeutic benefits in conditions such as corneal neovascularization [138].

Collectively, dissolvable microneedle platforms offer a noninvasive, tunable, and effective strategy for drug delivery to both anterior and posterior segments of the eye.

### 5.3. Personalized and Self-Medication Technologies

Technological advancements have catalyzed the emergence of personalized and point-of-care (POC) therapeutic strategies in ophthalmology. Among these, wearable contact lenses integrated with biosensors and wireless communication modules are being actively developed to enable continuous monitoring of ocular biomarkers, such as intraocular pressure, glucose, and electrolyte levels. The increasing number of patents related to biosensing contact lenses reflects the growing interest in multifunctional ocular devices with diagnostic and therapeutic capabilities.

Drug-eluting contact lenses (DECLs) offer customization potential for patient-specific therapeutic regimens, including the co-delivery of multiple drugs and personalized release kinetics tailored to the type and severity of ocular disease. These systems aim to combine vision correction with targeted pharmacotherapy, adapting both dosage and release profiles to individual needs. However, comprehensive clinical validation is still required before widespread implementation can be achieved [31].

A notable example is the microfluidic contact lens developed by Zhichang Du et al., which incorporates micropumps and microchannels into a stretchable, optically transparent hydrogel. This platform utilizes a pressure-driven release mechanism, allowing programmable drug delivery for acute and chronic treatments. In addition, blink-induced tear film motion enhances uniform drug distribution. The lens reservoir supports the encapsulation of small-molecule and macromolecular drugs for sustained release durations [143].

Parallel advances have also been made in diagnostic contact lenses. Xing Yang et al. developed UV-curable, hydrophilic, and flexible biomaterials for fabricating contact lenses with capillary microchannels and reservoirs containing chemical sensing substrates. These lenses generate colorimetric responses to tear biomarkers, such as glucose, chloride, and urea, with RGB image analysis enabling quantitative detection. In vitro, hydrogel-based ocular models validated the system’s sensitivity, comfort, and diagnostic functionality [144].

Together, these innovations underscore the transformative potential of polymeric materials and nanotechnology in advancing ophthalmic drug delivery and biosensing diagnostics in alignment with the broader goals of translational research and precision medicine.

## 6. Biopolymer-Based Contact Lenses for Ocular Drug Delivery

Numerous ocular disorders negatively impact visual function and quality of life. Among the most prevalent is conjunctivitis, a condition often characterized by excessive lacrimation and inflammation. Dry eye syndrome is another widespread disease primarily associated with insufficient tear production, which leads to ocular surface irritation, discomfort, and, in severe cases, visual impairment.

More serious pathologies include glaucoma, a neurodegenerative disease associated with progressive optic nerve damage, which—if left untreated—may result in irreversible blindness [145,146]. Other conditions with a significant global burden include age-related macular degeneration (AMD), diabetic macular edema (DME), uveitis, and cytomegalovirus (CMV) retinitis [147]. While various pharmacological and surgical interventions exist, efficient and targeted drug delivery to ocular tissues remains a formidable challenge due to physiological barriers and the limited bioavailability of conventional formulations.

Biodegradable polymers, such as poly(lactic-co-glycolic acid) (PLGA), polycaprolactone (PCL), chitosan, and hyaluronic acid, have gained increasing attention as drug carriers in ophthalmic applications because of their controlled degradation, low toxicity, and capacity for sustained release. These materials can be engineered to release therapeutic agents over durations ranging from a few days to several months, depending on factors such as polymer molecular weight, composition, and crosslinking density [119,148].

For example, PLGA-based delivery systems have been shown to sustain drug release for up to six months, making them particularly suitable for treating chronic conditions, such as age-related macular degeneration (AMD) or diabetic retinopathy, where frequent intravitreal injections are a significant clinical burden [149]. Recent studies confirm that biopolymer-based nanoparticles, hydrogels, and inserts can improve drug penetration, enhance retention time, and reduce dosing frequency, improving therapeutic outcomes and patient compliance [150].

In contact lens-mediated delivery, biodegradable coatings and embedded reservoirs offer additional benefits for the anterior segment. For instance, contact lenses modified with polydopamine (PDA) coatings have demonstrated effective anti-inflammatory drug release while maintaining optical clarity and mucoadhesiveness [3]. Other lens-integrated systems utilize chitosan or HA-based nanogels to deliver drugs for dry eye syndrome and post-surgical recovery [151].

### 6.1. Corneal Disorders

Progress in ocular biomaterials has significantly advanced the development of biopolymer-based therapeutic contact lenses for treating corneal diseases. Because of such CLs’ biocompatibility, biodegradability, mucoadhesive properties, and ability to encapsulate both hydrophilic and hydrophobic agents, natural and semi-synthetic polymers such as chitosan, collagen, bovine serum albumin (BSA), and cellulose derivatives are increasingly integrated into lens matrices, coatings, and drug reservoirs. These materials support sustained and localized drug release and promote epithelial regeneration, anti-inflammatory action, and antibacterial protection at the ocular surface. Researchers are actively exploring nanocarriers [152,153,154], hydrogels [155,156,157], contact lenses [158,159,160], and ocular implants [161,162,163] to enhance drug retention and bioavailability at the corneal surface. Among these, contact lenses have emerged as an up-and-coming platform.

Sun et al. developed ROS-responsive contact lenses co-loaded with levofloxacin and diclofenac, which exhibited minimal drug leakage during storage and provided inflammation-responsive release over 7 days. To enhance epithelial healing, the lenses demonstrated vigorous antibacterial activity and inhibited biofilm formation in bacterial keratitis models, particularly when combined with autologous serum [164].

Ye et al. engineered collagen-based hydrogel lenses using a two-step orthogonal crosslinking method, yielding optically clear, mechanically robust, and bioactive materials. This platform enabled stabilizer-free delivery of acidic fibroblast growth factor (aFGF) and showed therapeutic efficacy in alkali-induced corneal burns [165].

A bioinspired lens composed of cellulose nanocrystals (CNCs) and poly(hydroxyethyl methacrylate) (PHEMA), modified with cerium oxide nanoparticles (CeOx), was reported by Zhao et al. The material mimicked the native corneal structure and exhibited ROS-scavenging activity, promoting epithelial repair while maintaining transparency and cell compatibility [166].

Wang et al. utilized layer-by-layer electrostatic assembly of vancomycin-loaded chitosan nanoparticles and heparin to fabricate transparent, antibacterial contact lenses. The system was effective in both in vitro assays and a rabbit keratitis model [167].

Yin et al. investigated porous BSA-based contact lenses loaded with kaempferol, which exhibited sustained anti-inflammatory and antiangiogenic activity, reducing neovascularization in an alkaline corneal injury model [168].

Saleem et al. formulated thiolated chitosan nanoparticles containing tobramycin embedded into contact lenses. The resulting system extended drug release to 21 h—compared to 8 h for standard eye drops—and demonstrated good ocular tolerability [169].

Finally, Sadeghi et al. evaluated chitosan-coated therapeutic lenses to treat and prevent Acanthamoeba keratitis, reporting both prophylactic and therapeutic efficacy in vitro [170].

### 6.2. Bacterial, Fungal, and Viral Keratitis

Khan et al. [171] developed multifunctional therapeutic contact lenses using sonochemical surface modification. The coating—composed of tobramycin, gallic acid, and phytochemical-functionalized zinc oxide nanoparticles—provided antimicrobial and antifouling activity while enhancing surface wettability and wearer comfort. Although the biodegradability of the underlying lens matrix was not explicitly reported, such coatings are increasingly applied to hydrogel substrates based on biodegradable polymers, including chitosan and polycaprolactone (PCL), to ensure safe and gradual degradation upon prolonged ocular exposure [172].

In viral keratitis, particularly herpetic infections, conventional eye drops often demonstrate limited efficacy due to poor corneal penetration and short precorneal residence time. To address these challenges, Varela-Garcia et al. [173] employed molecular imprinting to fabricate contact lenses with a selective affinity for acyclovir and valacyclovir. Computational modeling guided the optimization of hydrogel monomer composition to enhance drug–polymer interactions. While the specific formulation was not identified as biodegradable, molecular imprinting techniques have recently been adapted with biodegradable polymer matrices, such as PLGA and gelatin, expanding their potential for sustained and environmentally degradable ocular drug delivery [174]. The lenses developed by Varela-Garcia et al. achieved controlled valacyclovir release over 10 h and demonstrated promising therapeutic potential for the localized treatment of recurrent herpetic keratitis.

### 6.3. Noninfectious Keratitis and Postoperative Inflammation

The rising number of ocular surgeries, including keratoplasty, cataract removal, and refractive procedures, has increased noninfectious keratitis and postoperative inflammatory complications. In this setting, drug-eluting contact lenses (DECLs) offer an effective platform for localized, sustained anti-inflammatory drug delivery, reducing the need for frequent eye drops and improving patient compliance during the postoperative period.

To address inflammation associated with keratoprosthesis, Carreira et al. [175] developed transparent drug-eluting films composed of chitosan, poly(vinyl alcohol) (PVA), and glyoxal as a crosslinker. The resulting system demonstrated good biocompatibility and sustained vancomycin release, suggesting its potential application as a biodegradable coating or insert in therapeutic contact lenses for inflammation control.

In another study, Jeencham et al. [176] fabricated single-use therapeutic contact lenses from chitosan blends and regenerated silk fibroin, incorporating diclofenac sodium as the active agent. The addition of silk fibroin enhanced the amorphous structure of the lens material and prolonged drug release, minimizing burst effects. These biopolymer-based lenses showed promise in preventing postoperative inflammation and reducing systemic side effects associated with repeated topical NSAID use.

### 6.4. Corneal Wound Healing

Delayed or incomplete healing of corneal injuries—from dry eye disease, corneal dystrophies, mechanical trauma, or limbal stem cell deficiency—can lead to serious complications, including infection, ulceration, and perforation. Clinical interventions include autologous serum therapy, drug-eluting bandage lenses, and amniotic membrane transplantation [177,178]. In recent years, biodegradable polymer-based contact lenses have emerged as promising alternatives to support epithelial regeneration, owing to their capacity for sustained drug release, biocompatibility, and resorbability in ocular environments.

Zhao et al. [179] developed gelatin-based hydrogel CLs capable of releasing rutin continuously for 14 days. In rabbit models, these lenses significantly accelerated corneal tissue regeneration without inducing adverse effects.

Expanding on multifunctional biomaterials, Yin et al. designed a porous film composed of bovine serum albumin (BSA), silver nanoparticles (Ag), and hyaluronic acid crosslinked into a stable matrix. When applied to a murine alkali burn model, the BSA/Ag/HA film demonstrated anti-inflammatory activity, enhanced re-epithelialization, and good ocular tolerability, supporting its potential as a therapeutic lens material [180].

Beyond passive drug release, Wu et al. introduced an electrically active bandage lens powered wirelessly via a micro-engineered flower-shaped chip capable of generating corneal surface electric fields. These fields mimicked endogenous bioelectric wound-healing cues, stimulating epithelial proliferation and migration without compromising lens transparency or visual acuity [181].

### 6.5. Keratoconus and Myopia

In disorders such as keratoconus and myopia, progressive weakening and disorganization of the corneal collagen matrix compromise the biomechanical integrity of the cornea. Although corneal cross-linking (CXL) is the current standard of care, conventional procedures often require epithelial debridement, resulting in postoperative pain, increased infection risk, and delayed healing [182,183,184].

Mun et al. [185] developed a noninvasive contact lens-based cross-linking system incorporating hyaluronan–rose Bengal (HA–RB) conjugate to overcome these limitations. The lens platform—fabricated using biodegradable hyaluronic acid derivatives—was activated on-demand by electrical stimulation, eliminating the need for epithelial removal. This strategy facilitated enhanced stromal penetration of the cross-linking agent. It ensured prolonged ocular residence time, offering a minimally invasive and patient-friendly alternative for biomechanical stabilization in keratoconus and early-stage myopia.

### 6.6. Glaucoma

Maintaining stable intraocular pressure (IOP) prevents optic nerve damage and disease progression in glaucoma. Conventional therapies based on topical eye drops often suffer from poor patient adherence, rapid drug elimination, and limited bioavailability. Responding to these limitations, contact lens-based delivery systems have gained increasing attention as drop-free platforms for sustained and targeted glaucoma management.

Baghban et al. [12] provided a comprehensive review of nanomaterial-integrated therapeutic contact lenses, highlighting their potential to improve IOP control through continuous release and enhanced corneal penetration. Building upon this concept, Xu Li et al. [186] designed a biomimetic contact lens inspired by the mechanical dynamics of fish fins. The lens incorporated deformable micro-reservoirs embedded within a flexible matrix capable of modulating drug release in response to posture-induced IOP fluctuations.

The lens structure is adapted passively to physiological changes in eye pressure, such as during sleep or physical activity, while maintaining continuous drug release. Although the authors did not explicitly describe the biodegradability of the matrix, the adaptive hydrogel base and microstructured design are compatible with ongoing efforts to implement biodegradable and self-regulating ocular materials for chronic conditions such as glaucoma and anterior uveitis.

### 6.7. Cataract Surgery and Postoperative Inflammation

Postoperative inflammation and infection are common complications following cataract surgery, particularly in patients with underlying ocular comorbidities. To reduce the reliance on frequent eye drop administration, biodegradable intraocular DDS has been developed to ensure sustained, localized therapy during the critical healing period.

Pinto et al. [187] designed a thin-film DDS composed of poly(lactic-co-glycolic acid) (PLGA), which was affixed to the haptics of intraocular lenses (IOLs). This fully biodegradable platform enabled the sustained release of dexamethasone for up to 30 days, effectively mitigating postoperative inflammation without additional intervention.

In a complementary approach, Subhash et al. [188] engineered a dual-drug biodegradable implant delivering dexamethasone and moxifloxacin, aligned with standard postoperative dosing schedules. In vivo testing in rabbit models of cataract surgery confirmed the system’s efficacy in controlling inflammation and preventing bacterial infection during the recovery phase.

### 6.8. Proliferative Ocular Diseases

Proliferative ocular conditions—such as post-glaucoma filtration scarring, proliferative vitreoretinopathy (PVR), and corneal fibrosis—significantly threaten long-term visual outcomes following surgical or inflammatory insult. Traditional treatment strategies often fail to achieve adequate drug localization and sustained therapeutic concentrations in affected tissues.

To address this challenge, Wu et al. developed a dual-layered contact lens composed of silicone and poly(vinyl alcohol) (PVA), loaded with pirfenidone (PFD)—a broad-spectrum antifibrotic agent with known activity in inhibiting TGF-β-mediated fibroblast proliferation [189,190]. The lens design significantly extended ocular residence time, lowering the required drug dosage while maintaining therapeutic efficacy.

Although silicone is non-biodegradable, PVA—a water-soluble, biocompatible polymer—contributes to controlled drug diffusion and integrates with biodegradable or hydrogel-based outer layers. The lens also exhibited high oxygen permeability and minimal protein adsorption, key features for maintaining ocular surface health during extended wear.

### 6.9. Ocular Cystinosis

Cystinosis is a rare systemic lysosomal storage disorder characterized by crystal deposition in ocular tissues, particularly the cornea, leading to photophobia, inflammation, and potential visual impairment. Management typically involves frequent topical administration of cysteamine, which suffers from short retention time and patient compliance issues.

To address photophobia, Dixon et al. [191,192] developed carbon black-infused contact lenses, which attenuated visible light transmission while maintaining lens transparency, effectively reducing photosensitivity in cystinosis patients. Additionally, incorporating vitamin E-enhanced UV protection and prolonged cysteamine release demonstrates dual functionality in drug retention and photoprotection.

Further advancements by Liu et al. [193] involved gold nanoparticle-embedded contact lenses, capitalizing on the strong cystine–gold affinity to improve therapeutic efficacy. While the base materials were not specified as biodegradable, such nanoparticle-functionalized platforms are increasingly being developed using biocompatible hydrogel matrices, including PVA and chitosan, to facilitate safe and extended treatment.

### 6.10. Uveitis

Uveitis treatment remains challenging because of the need for high drug doses, systemic side effects, and frequent instillations. Drug-eluting contact lenses have been explored to overcome these barriers for controlled corticosteroid and NSAID release.

Bengani et al. [194] engineered a ring-shaped polymeric reservoir integrated into metafilcon contact lenses, enabling sustained dexamethasone release over 7 days. The design minimized burst release and preserved optical clarity, demonstrating strong potential for topical corticosteroid-sparing.

Similarly, DiPasquale et al. [195] developed a shape-retaining hydrogel system that enabled 8-day controlled bromfenac delivery. The hydrogel, designed with “memory-based” polymeric behavior, showed consistent drug release without compromising lens comfort or biocompatibility.

These systems—though not always explicitly labeled as biodegradable—reflect the broader integration of biodegradable polymers, such as PLGA, hydrophilic copolymers, and surface-modified hydrogel coatings, in the design of long-acting ocular therapies for inflammatory diseases like uveitis.

### 6.11. Color Vision Deficiency (CVD)

Color vision deficiency (CVD) is a congenital visual disorder affecting the ability to distinguish specific color wavelengths—most commonly red and green—because of anomalies in cone photopigment function. Traditional management strategies rely on tinted spectacle filters, which may have limited optical precision and cosmetic appeal.

Recent advances have enabled the development of contact lenses incorporating red–green nanocomposites, designed to selectively filter problematic wavelengths using plasmonic nanomaterials and engineered metasurfaces [196,197,198]. These lenses demonstrated tunable spectral attenuation, improving color perception while maintaining transparency, ocular comfort, and cosmetic acceptability.

Although the optical components are primarily inorganic, they are increasingly integrated into soft hydrogel matrices—such as polyvinyl alcohol (PVA), silicone hydrogels, or emerging biodegradable copolymers—to enhance biocompatibility and allow potential coupling with drug-eluting or therapeutic functionalities. These multifunctional lenses represent a promising direction for visual correction and combined sensory and pharmacological interventions in ocular health.

These findings highlight the expanding role of therapeutic contact lenses across a wide range of ophthalmic indications, offering localized, sustained, and well-tolerated drug delivery options tailored to disease-specific needs.

An integrated overview of contact lens-based therapeutic strategies for selected ocular disorders is provided in Table 6.

## 7. Advanced Coating and Loading Strategies

### 7.1. Biopolymer-Based Coatings for Controlled Drug Release and Antimicrobial Protection

Natural polymers, such as starch, chitosan, alginate, and hyaluronate, have been employed to deliver antimicrobial compounds and control drug release from lens surfaces. For instance, starch-based hydrogels loaded with epigallocatechin gallate sustained release for 14 days and significantly inhibited Pseudomonas aeruginosa adhesion [199]. Layer-by-layer coatings composed of chitosan, alginate, and genipin further demonstrated effective microbial suppression and modulation of diclofenac release [200].

### 7.2. Asymmetric Drug Loading Strategies

Advanced drug delivery strategies increasingly exploit the ocular anatomy to improve therapeutic outcomes. One such approach is asymmetric drug loading, where the active compound is selectively localized on the posterior surface of the contact lens, in direct contact with the cornea. This design benefits from reduced tear turnover (ca. 30 min) in the post-lens tear film, resulting in prolonged drug residence time and enhanced bioavailability [201,202,203,204,205,206,207,208].

Malake Sarmout et al. [209] demonstrated the efficacy of this strategy using biopolymeric microparticles loaded with crystalline Rebamipide. These particles explicitly adhered to the corneal side of the lens and released the drug unidirectionally into the epithelium. In ex vivo porcine eye models, this method yielded a threefold increase in bioavailability compared to conventional eye drops (*p* < 0.001), underscoring the potential of directional, biopolymer-supported delivery systems.

Biopolymers, such as PLGA, chitosan, and alginate, are promising candidates for microparticle-based systems because of their biocompatibility, tunable degradation rates, and capacity for sustained release.

## 8. Biodegradable Polymers Beyond Contact Lenses: Intraocular Drug Delivery Systems

While therapeutic contact lenses have proven effective in improving ocular drug retention and patient compliance, intraocular lenses (IOLs) represent another promising platform for sustained drug delivery. Particularly in the postoperative management of cataract surgery, where repeated eye drop administration is challenging, biodegradable polymer coatings on IOLs can provide long-acting therapeutic effects directly within the eye.

Karamitsos et al. [210] developed thin, biodegradable films composed of PLGA and PCL copolymers. These were applied to the haptic arms of three-piece IOLs via spin-, spray-, and concave-coating methods. The films, loaded with dexamethasone, were sterilized using UV and plasma treatment to ensure microbial safety and structural integrity. Among the techniques evaluated, spin-coating produced the most uniform coatings with consistent release profiles sustained over several weeks.

Using PLGA and PCL allows for precise control over degradation rates and drug release kinetics, enabling customization to various therapeutic needs. This strategy can potentially replace or significantly reduce the need for postoperative eye drops, improving patient outcomes and adherence. Ongoing studies aim to scale manufacturing, assess long-term in vivo safety, and extend the platform to other drugs, including anti-VEGF agents and antibiotics [211].

This example illustrates the versatility of biodegradable polymers across ocular delivery platforms, reinforcing their central role in developing next-generation ophthalmic implants.

A comprehensive summary of translational applications of therapeutic ophthalmic lenses in preclinical and clinical contexts is presented in Table 7.

## 9. Technologically Enhanced Contact Lenses

### 9.1. Smart Contact Lenses

Smart contact lenses (throughout this review, the term “smart contact lenses” refers to contact lens systems enhanced with sensing, drug delivery, or digital functionalities; where appropriate, more specific terms, such as “sensor-integrated lenses” or “functionalized lenses”, are used to clarify the context) integrate microelectronic components and biosensors to extend functionality beyond traditional vision correction. These advanced systems are currently under intensive development for applications such as real-time health monitoring, controlled drug delivery, and augmented reality (AR) [227,228].

### 9.2. Diabetic-Eye Disease Monitoring and Therapy

Diabetic eye complications, such as diabetic retinopathy and cataracts, significantly impact visual health [229]. Smart lenses with glucose sensors, e.g., photonic microstructures or Fresnel-based bifocal systems, can continuously monitor glucose levels via tear film analysis using smartphone-based detection [230].

Alvarez-Rivera et al. developed hydrogel lenses functionalized with an aldose reductase inhibitor (epalrestat) to prevent lens opacification under hyperglycemic conditions [231]. Ross et al. encapsulated dexamethasone films in contact lenses for sustained retinal drug delivery, significantly reducing vascular leakage in diabetic retinopathy models [155].

### 9.3. Intraocular Pressure Monitoring Lenses

Functionalized contact lenses constitute novel ophthalmic biomaterials that integrate diagnostic and therapeutic capabilities. In glaucoma management, these lenses incorporate microsensors within biocompatible hydrogel matrices, enabling continuous intraocular pressure (IOP) monitoring without interfering with daily activities. Materials commonly used for such lenses include silicone hydrogels and HEMA-based polymers, selected for their oxygen permeability, flexibility, and long-term corneal compatibility.

Beyond pressure sensing, sensor arrays embedded in the polymer matrix can assess tear fluid composition, including electrolyte concentrations and pH. These properties support disease monitoring and highlight the versatility of contact lenses as multifunctional biomaterial platforms [51,162,232,233].

### 9.4. Intelligent Therapeutic Drug Delivery Platforms

Smart therapeutic contact lenses rely on stimuli-responsive polymeric systems, which release drugs in response to ocular or environmental cues, such as pH, temperature, or biomarkers like glucose and inflammatory cytokines [31,51,234,235]. These systems often employ hydrogels or biodegradable polymers such as chitosan, alginate, PEGDA, or PLGA, offering programmable release profiles and high biocompatibility.

Integrating such materials into lens substrates enables personalized, on-demand therapy with the potential to reduce dosing frequency and minimize systemic exposure. These advances place biomaterials at the core of intelligent ocular drug delivery systems.

### 9.5. Digital or Electronic Contact Lenses

Electronic contact lenses with AR capabilities represent an advanced application of functional biomaterials in ophthalmology. These devices combine transparent, flexible polymer substrates (e.g., PDMS, polyimide, PEG-based elastomers) with miniaturized microelectronic components, enabling the projection of digital information directly onto the retina.

To preserve comfort and ocular health, the substrate materials must exhibit oxygen permeability, tear film compatibility, and mechanical softness, qualities met by engineered biomaterials. Their integration with biosensing modules opens new directions in real-time physiological monitoring, including glucose sensing or intraocular pressure detection.

Despite significant progress, material–device integration challenges remain, including power management, thermal dissipation, and long-term in vivo stability [51,236,237,238,239].

### 9.6. Clinical Application of Continuous IOP Monitoring

Continuous IOP monitoring, particularly during sleep, represents a longstanding challenge in glaucoma care. A significant breakthrough was achieved with the FDA-approved Triggerfish^®^ smart contact lens (SENSIMED, Etagnières, Switzerland), which enables 24 h, noninvasive IOP monitoring, including during nocturnal periods [240]. Such innovations illustrate the practical potential of smart contact lenses in revolutionizing glaucoma diagnostics and long-term disease management.

## 10. Outlook

**Figure 6:**
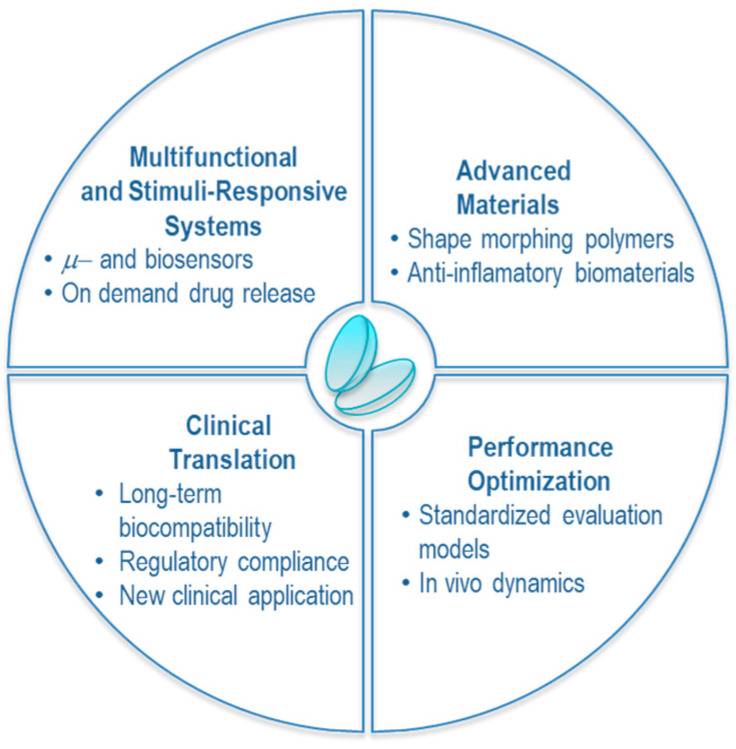
Key challenges and opportunities for DECLs.

The past decade has witnessed remarkable advancements in the field of DECLs, especially in developing biodegradable, innovative, and multifunctional systems tailored for targeted ocular drug delivery. Several opportunities and challenges define the roadmap as these innovations transition from preclinical evaluation to clinical consideration (Figure 6).

One key direction lies in integrating multifunctional components, such as biosensors and therapeutic reservoirs, within a single lens platform. Smart contact lenses capable of simultaneous diagnosis and therapy (theranostics), such as real-time glucose or intraocular pressure monitoring coupled with on-demand drug release, represent a transformative leap in ocular healthcare. However, such devices require optimization of power sources, wireless communication, and biocompatible microelectronics, which remain formidable engineering challenges.

The development of stimuli-responsive drug delivery systems continues to evolve, with an increasing focus on asymmetric loading, microfluidic architectures, and ROS- or pH-triggered release mechanisms. These technologies offer precise spatial and temporal control over pharmacokinetics and could be tailored for both anterior and posterior segment diseases. Future work should prioritize standardized evaluation models under dynamic tear flow and blinking conditions that simulate in vivo performance.

From a material science perspective, there is growing interest in 4D-printed, shape-morphing polymers and semi-synthetic biomaterials with intrinsic anti-inflammatory or regenerative properties. Such substrates may combine mechanical flexibility, oxygen permeability, and on-demand degradation profiles, aligning with the specific needs of ophthalmic therapy.

Clinically, the success of DECLs will depend on their ability to meet regulatory, economic, and patient-centric criteria. This includes long-term biocompatibility studies, manufacturing scalability, and integration with standard ophthalmic workflows. Furthermore, expanding the application of contact lens platforms into non-vision-corrective domains, such as post-surgical drug delivery, prophylaxis of neovascularization, or treatment of rare metabolic diseases, like ocular cystinosis, may broaden their therapeutic relevance.

Finally, advances in personalized medicine and AI-driven diagnostics could synergize with DECL platforms, enabling the design of individualized contact lenses tailored to a patient’s ocular pathology, pharmacogenomics, and wear patterns.

In conclusion, DECLs represent a rapidly maturing technology with the potential to shift the paradigm of ocular drug delivery. Continued interdisciplinary collaboration among material scientists, chemists, engineers, and clinicians will be essential to realize their clinical impact fully.

## 11. Clinical Translation and Future Implementation

Despite the promising preclinical outcomes demonstrated by various drug-eluting contact lens systems, their clinical translation remains a complex and multi-phase process. A significant challenge is ensuring long-term biocompatibility, mechanical stability, and predictable drug release kinetics under real-life ocular conditions, including blinking tear turnover and individual variability in corneal permeability. Regulatory frameworks, particularly those defined by the FDA and EMA, will require comprehensive data on toxicology, material degradation profiles, and interaction with existing ophthalmic treatments before any therapeutic lens reaches routine clinical use. Moreover, the manufacturability and scalability of advanced DECL technologies—such as those integrating biosensors, stimuli-responsive systems, or microfluidic components—must meet industrial standards for cost-efficiency, reproducibility, and shelf stability. Patient-centric factors, including comfort, ease of handling, wear compliance, and potential visual disturbances, will also play a decisive role in their acceptance. Clinical trials should focus on comparative effectiveness between DECLs and standard eye drops, especially for chronic conditions such as glaucoma, uveitis, or diabetic retinopathy. Multidisciplinary collaboration among chemists, biomedical engineers, clinicians, and regulatory experts will be critical to bridging the translational gap and advancing these innovative systems from bench to bedside.

## 12. Conclusions and Perspectives

In recent years, drug-eluting contact lenses have evolved from a promising concept into a multidisciplinary research frontier, poised to redefine ocular drug delivery. By combining vision correction with localized pharmacotherapy, DECLs offer advantages over conventional methods, including increased bioavailability, reduced dosing frequency, and improved patient compliance.

This review has outlined recent progress in material science, nanotechnology, and biomedical engineering that supports the development of DECLs. Notable innovations include asymmetric drug loading, stimuli-responsive hydrogels, microfluidic architectures, and biosensor integration. Together, these advances have broadened the functional scope of contact lenses, extending their use beyond vision correction into therapeutic and diagnostic domains.

Despite these promising developments, several translational challenges remain. Key among them is the need for the following:

- Robust preclinical models that simulate dynamic ocular environments.
- Standardized protocols for evaluating long-term safety, biocompatibility, and degradation.
- Scalable, cost-effective manufacturing workflows compliant with regulatory standards.
- Customizable designs tailored to the patient’s ocular anatomy, pharmacogenomic profile, and therapeutic timeline.

Future efforts should also focus on the regulatory harmonization of drug–device combination products, incorporating feedback from the FDA and EMA pathways. Furthermore, wireless integration, communication, and AI-driven diagnostics into contact lens platforms may catalyze the development of real-time, personalized ophthalmic care systems.

In conclusion, DECLs represent a paradigm shift in ophthalmology, potentially unifying diagnostics, drug delivery, and vision enhancement in a single, wearable platform. Ongoing interdisciplinary collaboration will be essential to fully realize their clinical potential and facilitate the transition of these technologies from the research laboratory to clinical practice.

## Figures and Tables

**Table 1:** Comparison of the regulatory classification of contact lenses between the United States Food and Drug Administration (FDA) and the European Medicines Agency (EMA) under the Medical Device Regulation (MDR 2017/745).

Criteria	FDA (USA)	EMA/MDR (EU)
Regulatory status	Medical device—Class II or III (21 CFR 886.5925).	Medical device—Class IIa (per MDR 2017/745)
Classification basis	Clinical use, wearing time, material composition.	Clinical use, wearing time, mechanism of action
Contact lens categories	• Corrective; • Therapeutic; • Cosmetic; • Orthokeratology.	• Corrective; • Therapeutic; • Cosmetic; • Orthokeratology.
Drug-eluting therapeutic lenses	Combination product (drug–device) requires pharmaceutical and device-specific data. Regulated jointly by CDRH and CDER.	Combination products, must comply with MDR and relevant medicinal product legislation (e.g., Directive 2001/83/EC)
Pre-market requirements	Evaluation of safety, efficacy, PK/PD, biocompatibility, GMP compliance.	Clinical evaluation, CE documentation, ISO compliance, notified body assessment
Wearing time classification	Daily wear (DW)—<24 h Extended wear (EW)—>24	Based on duration and intended use; >24 h requires additional evaluation under MDR
Material compliance	Biocompatibility per ISO 10993 and FDA guidance documents.	Biocompatibility per ISO 10993 and MDR Annex I (General Safety and Performance Requirements)

**Table 2:** Recent progress in drug-eluting contact lenses.

Title	Keywords/Aims	Ref.
Recent Advancements in Nanomaterial-Laden Contact Lenses for Diagnosis and Treatment of Glaucoma, Review and Update	Glaucoma; Contact Lenses; Drug Delivery System; Diagnosis; Treatment	[12]
Review Article Testing Drug Release from Medicated Contact Lenses: The Missing Link to Predict In Vivo Performance	Drug-Eluting; Contact Lens; In Vitro Release Tests; In Vivo Release Rate Specifications; Therapeutic Response; In Vitro–In Vivo Correlations	[22]
Advancements in the chemistry of contact lenses: innovations and applications	Contact Lens Chemistry; Polymer Materials; Hydrogels and Silicone Hydrogels; Smart Contact Lenses; Antimicrobial Coatings	[14]
The Promise of Drug-Eluting Contact Lenses	An Overview of Drug-Eluting Contact Lens Technologies and Lenses That Are in Preparation	[28]
Review of Approaches for Increasing Ophthalmic Bioavailability for Eye Drop Formulations	Bioavailability; Drug Delivery; Eye Drops; Nanoparticles; Permeability Enhancers	[29]
Contact Lenses as Ophthalmic Drug Delivery Systems: A Review	Contact Lenses; Ophthalmic Drug; Polymeric Support; Ocular Drug Delivery	[30]
Next-Generation Contact Lenses: Towards Bioresponsive Drug Delivery and Smart Technologies in Ocular Therapeutics	Contact Lens; Drug-Eluting; Ocular Surface; Biosensing; Ocular Therapeutics; Drug Delivery	[31]
Controlled Drug Delivery Systems: Current Status and Future Directions	Controlled Release Dosage Forms; Pharmacokinetics; Nano-Drug Delivery; Smart and Stimuli-Responsive Delivery; Intelligent Biomaterials	[32]
In Vivo Drug Delivery via Contact Lenses: The Current State of the Field from Origins to Present	Contact Lens; Drug Release; In Vivo; Ophthalmic Therapy; Therapeutic Contact Lens	[33]
Sustained Bimatoprost Release using Gold Nanoparticles Laden Contact Lenses	Contact Lens; Animal Studies; Bimatoprost; Gold Nanoparticles; Sustained Drug Delivery	[34]
BCLA CLEAR—Medical Use of Contact Lenses	Therapeutic Contact Lens; Bandage Lens; Scleral Lens; Irregular Astigmatism; Aphakia; Ocular Surface Disease	[35]
Soft Contact Lenses as Drug Delivery Systems: A Review	Contact Lenses; Drug Delivery; Drug-Controlled Release; Drug Delivery Systems Based on Contact Lenses in Ophthalmic Therapies	[36]
Drug Delivery to the Anterior Segment of the Eye: A Review of Current and Future Treatment Strategies	Optimizing Ophthalmic Drug Delivery by Achieving High Drug Concentrations with a Prolonged Duration of Action that is Convenient for Patient Administration	[37]
Considerations for Polymers Used in Ocular Drug Delivery	Controlled Release; Drug Delivery; Hydrogel; Ocular Biomaterials; Ocular Implants; Ophthalmic Delivery; Polymer	[38]
Lab-on-a-Contact Lens: Recent Advances and Future Opportunities in Diagnostics and Therapeutics	Bioelectronics; Biosensors; Contact Lens; Diagnostics; Integrated Systems; Personalized Healthcare; Therapeutics; Wearable Electronics	[39]
Drug Delivery Strategies and Biomedical Significance of Hydrogels: Translational Considerations	Hydrogels; Drug Delivery; Therapeutic Interventions; Clinical Trials; Translation; Biomedical Perspectives; Contact Lenses; Wound Management; Tissue Engineering	[40]
Updates on Biodegradable Formulations for Ocular Drug Delivery	Biodegradable Drug Delivery; Ocular Drug Delivery; Biodegradable Polymers; Nanoparticle Drug Delivery; Polymeric Micelles; Liposomes; Hydrogels; Biodegradable Implants	[41]
Therapeutic Applications of Contact Lens-Based Drug Delivery Systems in Ophthalmic Diseases	Drug Delivery; Contact Lens; Ophthalmic Diseases; Polymer Material	[42]
Contact Lens as Drug Delivery System for Glaucoma Treatment: A Review	Glaucoma; Intraocular Pressure; Gold Nanoparticles (GNPs); Timolol; Drug Delivery; Bioavailability	[43]
Development of Corneal Contact Lens Materials and Current Clinical Application of Contact Lenses: A Review	Drug-Eluting Contact Lenses: Progress, Challenges, And Prospects	[44]
Contact Lenses for the Treatment of Ocular Surface Diseases	Bandage Contact Lens; Dry Eye; Ocular Surface Disease; Prosthetic Contact Lens; Rigid Gas Permeable; Scleral Contact Lens; Stevens–Johnson Syndrome; Therapeutic Contact Lens	[45]
Contact Lenses as Ophthalmic Drug Delivery Systems—The Future of Treatment for Ocular Infection and Injuries—A Review	Therapeutic Contact Lens; Antibiotic-Releasing Contact Lens; Contact Lens Application	[46]
Review Applications of Hyaluronic Acid in Ophthalmology and Contact Lenses	Hyaluronic Acid; Contact Lenses; Ophthalmology	[47]
Role of Therapeutic Contact Lenses in the Management of Corneal Disease	Keratoconus; Ocular Surface Disease; Scleral Lens; Therapeutic Contact Lens	[48]
Contact Lenses as an Ophthalmic Drug Delivery System	Contact Lenses; Ophthalmic Drug; Polymeric Support; Ocular Drug Deliver	[49]
Pharmaceutical-Loaded Contact Lenses as an Ocular Drug Delivery System: A Review of Critical Lens Characterization Methodologies Regarding ISO Standards	Ocular Drug Delivery; Therapeutic Contact Lens; Characterization Techniques; Physical Properties; Chemical Properties; ISO Standards	[50]
Ocular contact lenses: smart materials for biomedical applications	Contact Lenses; Silicone Acrylate-Based Polymers; Optical Disorders; Therapeutic Lens; Biomaterials	[51,52]
From Vision Correction to Drug Delivery: Unraveling the Potential of Therapeutic Contact Lens	Therapeutic Contact Lens; Contact Lenses; Drug Release; Drug Stability; Ocular Surface Disorders; Vision Correction	[53]
Carbohydrate Polymers, Polymeric Nano Drugs, and Nanoparticles Are Used for Advanced Drug Delivery and Therapeutics in Ocular Diseases	Carbohydrate Polymers; Polymeric Nano-Drugs; Nanoparticles; Contact Lenses	[54]
Polymeric Membranes in Contact Lens Technology for Glaucoma Treatment: Breakthroughs, Obstacles, and Emerging Opportunities	Contact Lenses; Drug Delivery; Glaucoma; Hydrogel, Nanoparticle; Polymers	[55]
Microfluidic contact lens: fabrication approaches and applications	Microfluidic Contact Lens	[56]
Recent Advances in New Copolymer Hydrogel-Formed Contact Lenses for Ophthalmic Drug Delivery	The Use of HEMA, MAA, DMA, NYP, EGDMA, TRIS, and PDMS in Therapeutic Contact Lenses; The Advantages and Disadvantages of Each Material in Tailoring the Drug Release Rate for Different Encapsulated Payloads, With Particular Emphasis on Their Impact on Therapeutic Efficacy	[57]
Emerging Role of Hydrogels in Drug Delivery Systems, Tissue Engineering and Wound Management	Hydrogel; Stimuli-Responsive; Polymeric Hydrogel Nanoparticles; Drug Delivery Systems; Wound Dressing Materials; Tissue Engineering Scaffolds; Modified Contact Lens	[58]
Drug-Modified Contact Lenses—Properties, Release Kinetics, and Stability of Active Substances with Particular Emphasis on Cyclosporine A: A Review	Therapeutic Contact Lenses; Polymer Matrix; Drug Stability; Mechanic Parameters; Cyclosporine Stability; Drug Delivery Systems	[59]

**Table 3:** Overview of synthetic polymers applied in ophthalmic biomedical systems.

Monomer	Polymer Properties, Advantages/Limitations	Ref.
	Polyethylene glycols	
Ethylene Glycol (EG)	PEG (poly(ethylene glycol)): Water-soluble and highly biocompatible; exhibits faster degradation than other synthetic polymers; commonly used for surface modification and drug conjugation	[68,69,70]
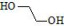		
	Polyvinyl alcohols	
Vinyl Alcohol (VA)	PVA (poly(vinyl alcohol)): Characterized by slow degradation under physiological conditions; typically synthesized using harsh organic solvents	[71]
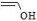		
	Polyesters	
Glycolic Acid (GA)	PGA (poly(glycolic acid)): Exhibits rapid hydrolytic degradation; limited mechanical strength; rarely used alone because of brittleness	[2,72,73]
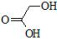		
Lactic Acid (LA)	PLA (poly(lactic acid)): Synthesized from renewable natural resources; good mechanical strength and processability; limited impact resistance; slow and incomplete biodegradation under physiological conditions	
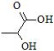		
GA + LA	PLGA (poly(lactic-co-glycolic acid)) Biocompatible and FDA-approved copolymer; controlled and tunable degradation rate; most applied polymer in ocular drug delivery platforms	
ε-Caprolactone (CL)	PCL (poly(caprolactone)): Biodegradable, hydrophobic, excellent biocompatible, semi-crystalline polyester, mechanical flexibility, slow degradation profile, easy to modify, inexpensive, widely explored in ophthalmic drug delivery, not specifically FDA-approved for ophthalmic use	[41,74,75]
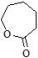		
Ortho ester (OE)	POE (poly(ortho ester)): Undergo surface erosion during degradation; limited data available on their application in ocular drug delivery systems	[76]
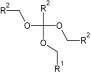		
	Polymethacrylates	
Methyl methacrylate (MMA)	PMMA (poly(methyl methacrylate)): Well-established ophthalmic polymer; cost-effective and resistant to UV radiation and environmental exposure; non-biodegradable; limited chemical and thermal resistance; low oxygen permeability	[77,78]
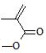		
2-Hydroxyethyl Methacrylate (HEMA)	pHEMA (poly(2-hydroxyethyl methacrylate)): Hydrophilic and water-absorbing material; biocompatible but non-biodegradable; rigid when dry and soft, flexible when hydrated; exhibits poor mechanical strength; capable of hydrolysis, ionization, and hydrogen bonding; suitable for modulating slow drug release	[79,80,81]
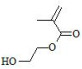		
2-(Dimethylamino)ethyl Methacrylate (DMAEM)	PDMAEM (poly-2-(dimethylamino))ethyl methacrylate: Methacrylate-based polymer used in ocular hydrogels, nanoparticle carriers, micelles, and implants; chemically stable but incompatible with strong acids, bases, and oxidizers; prone to auto-polymerization and degradation upon exposure to air, moisture, or light	
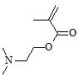		
	Polyolefins	
Acrylic Acid (AA)	PAA (poly(acrylic acid)): Highly water-soluble and mucoadhesive polymer; biodegradable, yielding acidic degradation products; widely explored for controlled ocular drug delivery	[82]
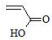		
	Dendrimers	
Ethylenediamine	PAMAM (poly(amidoamine)): Highly branched dendrimer with numerous reactive surface groups; enables facile chemical functionalization; not currently FDA-approved for ophthalmic applications	[83,84]
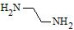		

**Table 4:** Structural units of biopolymers applied in ocular drug delivery systems.

Polymer	Structure	Characteristics	Ref
Polysaccharide biopolymers			
Dextran	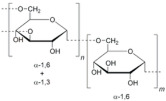	DEX: Biocompatible, biodegradable, and hydrophilic biopolymer; capable of forming hydrogels; FDA-approved and commonly used in ophthalmic eye drop formulations.	[86,87]
Cellulose	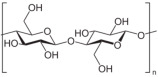	CEL: Biocompatible and biodegradable via enzymatic degradation and hydrolysis; chemically reactive and amenable to conjugation; FDA-approved for ophthalmic applications.	[85]
Carboxymethylcellulose	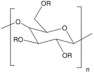	CMC: Biocompatible and hydrophilic linear polymer; an effective matrix for experimental biopolymer-based hydrogels and thin films enabling sustained local drug release.	[85,88,89]
Chitosan	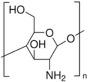	CHI: Mucoadhesive, biocompatible, exhibits antimicrobial and anti-inflammatory properties, enhances drug retention on the ocular surface. Poor solubility at neutral and alkaline pH; batch-to-batch variability in molecular weight and degree of deacetylation affects stability and reproducibility.	[90,91]
Hyaluronic acid	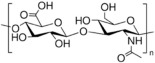	HA: Naturally occurring, biocompatible, and biodegradable polysaccharide with high water retention capacity; exhibits viscoelastic and mucoadhesive properties; widely used in ocular formulations to promote wound healing and lubrication.	[92,93,94]
Pullulan	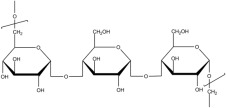	PUL: Biocompatible, nonionic, and biodegradable polysaccharide; water-soluble and stable across a wide range of temperatures and pH; insoluble in most organic solvents; oxygen-impermeable, viscosity-enhancing, and easily processed for ocular formulations.	[95,96,97]
Guar gum	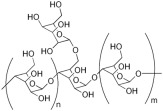	GG (Galactomannan): Biocompatible, water-soluble, and mucoadhesive polysaccharide; nonionic and hydrolytically degradable; exhibits strong swelling capacity and increases viscosity; FDA-approved for ophthalmic use. Limited solubility in alcohols and organic solvents; unstable in solution over time.	[98]
Protein biopolymers			
Collagen	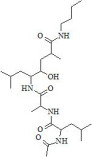	COL: Biocompatible and enzymatically degradable structural protein. The primary sequence motif repeats Gly–X–Y, where Gly = glycine (every third residue), X = usually proline, and Y = usually hydroxyproline or hydroxylysine. Relatively easy to process and widely available from animal sources (e.g., bovine, porcine), recombinant collagen offers a safer and more sustainable alternative via plant and yeast expression systems.	[99,100,101]
Gelatin	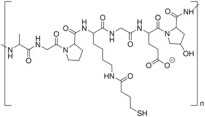	GEL: Biocompatible, biodegradable, and water-soluble protein derived from collagen; forms gel and increases viscosity; cost-effective and widely available; exhibits lower gelation temperature and higher aqueous solubility than native collagen.	
Other biopolymers			
Poly(dopamine)	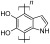	PDA: Biocompatible and low-toxicity polymer formed via oxidative polymerization of dopamine; extensively explored in drug delivery for its strong adhesion to diverse surfaces; widely used in developing biofunctional coatings and nanostructures.	[102]

**Table 5:** Emerging biopolymeric materials for ocular drug delivery: properties, applications, and research status.

Polymer	Application in Ophthalmology	Research Status	Ref.
Silk fibroin	SF: Used in transparent corneal scaffolds and drug-loaded therapeutic contact lenses	Emerging clinical interest	[110]
Poly(hydroxy alkanoates)	PHA/P3HB: Controlled drug release in ocular implants and corneal patches	Preclinical and translational studies	[112]
Poly(glycerol sebacate)	PGS: Flexible biodegradable substrate for ocular implants and hydrogels	Experimental phase	[111]
Poly(trimethylene carbonate)	PTMC: Tested as a coating for intraocular lens systems	Investigational	[113]
Poly(ester amide)	PEA: Ocular drug carriers with tunable degradation for retinal delivery	Advanced preclinical development	[114]
Zwitterionic hydrogels	Z-HYD: Biofilm-resistant hydrogels for drug-eluting contact lenses	Proof-of-concept studies	[115]
Methacrylated hyaluronic acid	MeHA: Enhanced HA hydrogels for sustained drug release in CLs	In vitro/in vivo validation	[116]
Polysaccharide nanogels	PSNG: Nanogels for anterior and posterior segment drug delivery	Exploratory nanomedicine studies	[117]
Polydopamine derivatives	PDA-PEG, PDA-HA: ROS/pH-sensitive coatings for targeted ocular drug release	High potential, under development	[118]

**Table 6:** Overview of contact lens-based therapeutic platforms for selected ocular disorders.

Condition	Lens Type/Carrier	Active Agent	Mechanism of Action	Validation
Corneal wound healing	Gelatin hydrogel, HA/Pluronic^®^, BSA/Ag/HA, electric lenses	Rutin, HA, silver, e-stimulation	Anti-inflammatory, ROS scavenging, epithelial repair	In vivo (rabbit, mouse)
Keratoconus and myopia	HA-RB conjugate lens (photoactivated)	Rose Bengal	Collagen photo-crosslinking without epithelial disruption	In vivo (preclinical)
Glaucoma	Microstructured lenses, DEX-ring, bromfenac-loaded hydrogel	Dexamethasone, bromfenac	Sustained/pressure-responsive delivery	In vivo (rabbit)
Cataract (postop)	PLGA-coated IOLs, dual-drug implants (DEX + MOX)	Dexamethasone, moxifloxacin	Anti-inflammatory + antibacterial postop protection	In vivo (rabbit)
Proliferative ocular diseases	Silicone-PVA layered lenses	Pirfenidone	Antifibrotic, extended tear residence	In vivo (rabbit)
Ocular cystinosis	Cysteamine + carbon black or gold NP lenses	Cysteamine, vitamin E, gold NP	Prolonged release, UV protection, cystine binding	In vivo, in vitro
Uveitis	DEX-ring, bromfenac hydrogel lenses	Dexamethasone, bromfenac	Sustained anti-inflammatory release	In vivo (rabbit)
Color vision deficiency (CVD)	Nanocomposite/metasurface/plasmonic lenses	Optical modulation	Wavelength filtering for enhanced color vision	In silico, prototyping

**Table 7:** Translational applications of therapeutic ophthalmic lenses in the treatment of vision-threatening disorders.

Ophthalmic Diseases	Techniques	Active Principle	Findings/Results	Ref.
Bacterial keratitis	Coating	Copper ions	Endows CLs with the ability to effectively inhibit biofilm formation	[212]
Bacterial keratitis	FRP free radical polymerization	EGCG epigallocatechin gallate	Sustained drug release over 14 days; significantly inhibits *P. aeruginosa* adhesion	[199]
Fungal keratitis	Nanocoatings	Gallic acid, tobramycin	Significant antimycotic, biofilm inhibition, and antifouling properties	[171]
Viral keratitis	Hydrogels based on HEMA, EGDMA, MAA, AIBN; molecular imprinting	Acyclovir, valacyclovir	Releases the drug in a sustained manner for 10 h	[173]
Noninfectious keratitis	DPF drug–polymer film	Vancomycin	Sustainably released for more than 8 h	[176]
Corneal wound healing	FRP free radical polymerization	Rutin	Sustained rutin release over 14 days; facilitates corneal wound healing	[179]
	FRP free radical polymerization	HA hyaluronic acid	Reduces ocular inflammation; supports corneal healing in preclinical models	[213]
	DPF drug–polymer film	HA, silver	Prolonged hyaluronic acid retention accelerates corneal healing	[180]
Glaucoma	Polyurethane film produced by solvent casting; soaking	Brimonidine tartrate	Prolonged drug release up to 14 days	[214]
Conjunctivitis	Lipid-based film—drug-loaded liposomes by hydration method; soaking	Besifloxacin hydrochloride	Biphasic release: initial burst + sustained (80% released in 10 h)	[215]
Ulcerative keratitis	Soaking	Ciprofloxacin hydrochloride, tobramycin	Antibacterial activity for 48 h	[216]
Corneal gene therapy	HEMA hydrogels; soaking	rAAV	Efficacy in transduction/triggering cell proliferation	[217]
Conjunctivitis	HEMA/CD hyaluronan; soaking	Diclofenac sodium	Therapeutic effect for conjunctivitis	[218]
Retinoblastoma	PEG-modified silicone; soaking	Roscovitine	Prolonged drug release	[219]
Acanthamoeba keratitis	Commercial hydrogel-based CLs; soaking	Voriconazole, diclofenac sodium	Sustained release, cell proliferation	[220]
	Commercial CLs based on silicone or HEMA hydrogels; soaking	Tetracaine, bupivacaine, ketotifen, diclofenac, flurbiprofen; loading of fatty acids (i.e., oleic acid, linoleic, linolenic acid)	Initial burst release: 30–90% (dependent on drug–lens system) followed by sustained release phase	[221]
Endophthalmitis after cataract surgery	Commercial foldable acrylic CLs; supercritical impregnation	Gatifloxacin	Improvement in impregnation yield	[222]
Posterior capsule opacification after cataract surgery	Commercial foldable acrylic CLs; supercritical impregnation	Methotrexate	Prolonged drug release for more than 100 days, inhibition of fibrosis	[222]
Ocular hypertension, glaucoma	Silicone CLs, implants based on Irgacure, EGDMA, DMA, NVP, siloxane, and HEMA, then embedded into silicone CLs; soaking	Bimatoprost, hyaluronic acid, timolol	High burst effect in drug release profiles	[223]
Glaucoma	Sil-DMA-HEMA	Timolol		[224]
Glaucoma	HEMA-DMA/GMA/Sil	Timolol		[225]
Proliferative ocular diseases	Drug–polymer film	Pirfenidone	Increased duration of pirfenidone	[189,190]
Ocular cystinosis	Nanoparticles	Gold NPs	Cystine binding for ocular cystinosis management	[193]
Uveitis	Drug–polymer film	Dexamethasone	Provides 7-day corneal anti-inflammatory activity and 5-day anterior uveitis suppression	[194]
Uveitis	Molecular imprinting	Bromfenac	Eight-day sustained release of bromfenac in vivo	[195]
Color vision deficiency	Metasurfaces	Metasurfaces	Spectral correction of misperceived pigments for color vision enhancement	[198]
Bacterial keratitis	Coating	Glycidyl methacrylate	Against MRSA with killing efficacy > 99.99%	[226]
Ocular inflammation-related disorders	PLGA: thin film	Dexamethasone	Provides >30-day sustained dexamethasone release with demonstrated efficacy	[187]

## Data Availability

Data sharing is not applicable.
